# Salinity Tolerance in Wheat: Mechanisms and Breeding Approaches

**DOI:** 10.3390/plants14111641

**Published:** 2025-05-27

**Authors:** Indrila Dey Traye, Nur Mohammod Oli, Xuelian Weng, Ke Li, Mohamed Suliman Eltyeb Suliman, Xiaoqian Guo, Guisheng Zhou, Guanglong Zhu, Yunji Xu

**Affiliations:** 1 Joint International Research Laboratory of Agriculture and Agri-Product Safety, The Ministry of Education of China, Yangzhou University, Yangzhou 225009, China; indriladeytraye@gmail.com (I.D.T.); 18118277525@163.com (X.W.); m.suliman@yzu.edu.cn (M.S.E.S.); g.zhu@yzu.edu.cn (G.Z.); 2 College for Overseas Education, Yangzhou University, Yangzhou 225009, China; hasanoly9@gmail.com (N.M.O.); gszhou@yzu.edu.cn (G.Z.); 3 Huaiyin Institute of Agricultural Sciences of the Xuhuai District of Jiangsu Province, Huaian 223001, China; lk2311586@163.com

**Keywords:** wheat (*Triticum aestivum* L.), salt-tolerant mechanism, morphological adaptation, physiological and biochemical adaptations, genetic management

## Abstract

High salinity and other abiotic stressors severely limit the productivity of wheat (*Triticum aestivum* L.). Wheat is a moderately salt-tolerant crop, and its salinity tolerance has been extensively studied due to the fact that it is one of the most essential food crops. It is essential to comprehend the mechanisms underlying salinity tolerance and create adaptable wheat types. In this paper, the morphological adaptations in wheat were first introduced under salinity stress, then the main physiological, biochemical and molecular reactions of wheat to salinity stress were summarized in detail. In addition, the advances in breeding approaches to salinity tolerance in wheat through germplasm evaluation, screening and gene editing were generally reviewed. Finally, proposals for further research or possible challenges in this process were also discussed. Our review will provide references for improving salt tolerance of wheat and for breeding salt-tolerant varieties.

## 1. Introduction

Salinity is one of the main abiotic stresses that have significant effects on crop productivity globally [1]. The two leading causes of soil salinity are natural (primary salinization) and human-made (secondary salinization). Seawater intrusion and the occurrence of parent materials and physical–chemical weathering of minerals are the major natural reasons for soil salinization [2]. The use of wastewater for crop irrigation caused be prolonged dryness, combined with heavy chemical fertilizers, is the main anthropogenic cause of soil salinization [3]. Sodium salts are the main constituents of many saline soils, and salinity affects around 800 million hectares of land [4].

Wheat (*Triticum aestivum* L.) is a staple crop that makes a substantial contribution to global food security. However, growing wheat is seriously threatened by soil salinization, which is made worse by poor irrigation techniques and climate change, particularly in dry and semi-arid areas [5]. A significant number of wheat-growing regions are harmed by salinity, which affects about 20% of the world’s irrigated acreage [6]. By report, with global wheat production losses related to salinity approaching 10–15 million metric tons yearly, it is projected that wheat yields can drop by as much as 50% under moderate saline levels (7–8 dS m^−1^) [7]. Thus, creating wheat cultivars that can endure salinity is essential to maintaining output in saline conditions. Studies have shown that salinity stress hinders plant growth by interfering with water intake and causing ion toxicity, oxidative stress, and nutritional imbalances [8,9]. For wheat to flourish in salt environments, it needs improved tolerance mechanisms [10]. Interfering with water intake and creating a high salinity level can negatively impact wheat growth and result in lower crop yields [11,12]. In order to sustain output in impacted areas, wheat needs improved resistance to salty conditions. Researchers and breeders can create wheat cultivars that can flourish in saline environments by comprehending the principles underlying salt tolerance and utilizing cutting-edge breeding procedures. These initiatives will be essential to maintaining the global wheat output and guaranteeing food security for future generations as the stresses of population expansion and climate change increase [13]. In order to overcome the difficulties caused by salt and preserve wheat’s resilience, it will be essential to combine traditional and modern breeding techniques. Enhancing wheat’s resistance to salinity is crucial for maintaining food security in areas that are prone to it. Wheat’s adaptation to salt stress will be improved by fusing cutting-edge breeding methods [14]. Ionic imbalance, osmotic damage, and the production of reactive oxygen species are all consequences of salinity stress that can seriously hinder the growth and development of plants [8,9].

As a significant cereal crop, wheat has evolved a number of defenses against the negative effects of salinity. Wheat plants have developed ways to reduce the net intake of sodium at the soil–root interface and its xylem loading in the roots in order to mitigate the detrimental effects of rising salt [4]. These strategies include controlling ion transport to preserve cellular equilibrium, modifying osmotic pressure by accumulating organic solutes, and triggering antioxidant defenses to lessen oxidative stress brought on by salt [11,12]. The goal of breeding strategies like gene editing and marker-assisted selection (MAS) is to improve these characteristics [15]. Salt-tolerant wheat cultivars can be produced by combining conventional and molecular breeding, providing answers for food security and agricultural production in areas affected by salt. Breeding initiatives have advanced the development of wheat varieties that can withstand salinity by utilizing both conventional techniques and contemporary technology like MAS, genetic engineering, and genomic selection (GS) [16]. Even with great advancements, creating wheat cultivars that can withstand high salt still presents difficulties. The intricacy of the salt tolerance characteristic, which is impacted by several genes and environmental interactions, is one of the main obstacles [17,18]. To overcome these obstacles, it will be essential to combine genetic technology, bioinformatics, and high-throughput phenotyping. For wheat to lessen the harmful effects of salt, it is essential to maintain low sodium levels in the shoot, which is accomplished via effective sodium exclusion mechanisms [4,19]. Therefore, in order to provide a theoretical basis for improving wheat salt tolerance and breeding salt-tolerant types, our review describes main morphological adaptations in wheat under salinity stress, highlights the physiological, biochemical and molecular reactions of wheat to salinity stress and the advances in genetic management for salinity tolerance, and finally, discusses the potential research in the future.

## 2. The Morphological Adaptations of Wheat Under Salinity Stress

**Figure 1:**
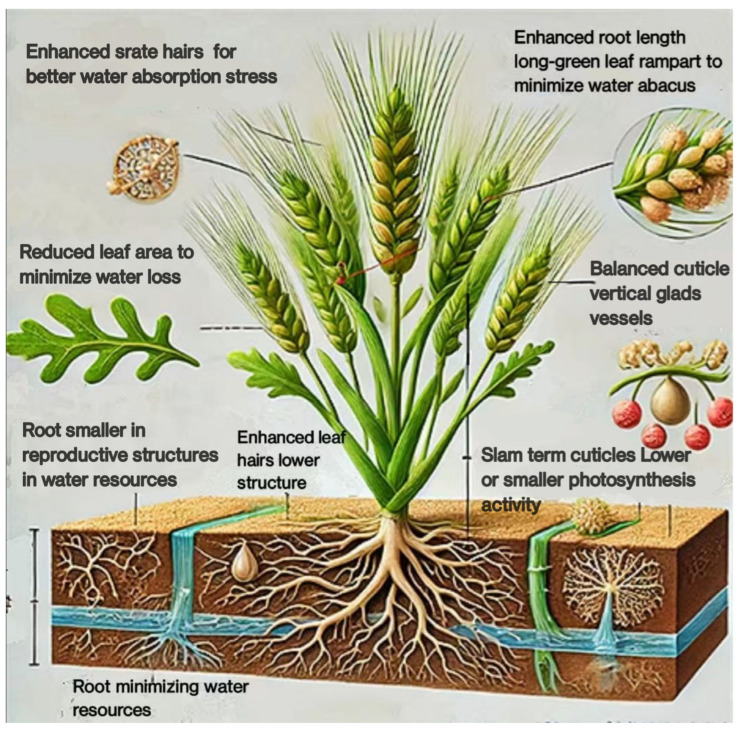
The morphological adaptations of wheat under salinity stress.

Particularly in dry and semi-arid areas, wheat is frequently subjected to salinity stress. Wheat growth, development, and yield are adversely affected by salinity stress because it upsets osmotic equilibrium, ion toxicity, and nutritional imbalance. At the cellular, tissue, and whole-plant levels, wheat displays a number of morphological modifications to address these issues [20]. In order to obtain water from lower soil layers, where saline levels may be lower, wheat plants frequently grow larger and deeper root systems. Increased surface area for water and nutrient uptake due to improved root hair growth helps lessen the impacts of salinity. Ion uptake is decreased and harmful ion accumulation in the shoot is avoided by thicker root cell walls and greater suberization (suberin deposition) in the endodermis. The terms “Enhanced root length” and “Root smaller in reproductive structures in water resources”, which are mentioned in Figure 1, indicate that the morphology of the roots has been adapted to increase water absorption and efficiency, particularly with stress. It most likely alludes to improved root growth and root hairs that boost the absorption of water and nutrients under stressful circumstances. In saline environments where water intake is restricted, wheat plants may decrease leaf area to minimize water loss through transpiration (Figure 1). In order to sustain turgor pressure under osmotic stress, leaves may thicken as a result of reduced cell growth and increased cell wall thickness. To save water and lessen the exposed surface area, some wheat cultivars roll their leaves [21].

Under salinity stress, wheat plants frequently block their stomata to minimize transpiration and water loss. Water conservation is aided by this, although photosynthesis and CO_2_ uptake may be restricted [22]. To reduce water loss, certain wheat cultivars produce smaller and fewer stomata. Phrases such as “Reduce leaf or water abrades” and “long-green leaf rampart to minimize water abacus” imply changes to the structure of leaves in order to minimize water loss (Figure 1). Changes in leaf size, shape, or surface characteristics may fall under this category. In order to improve water and nutrient uptake during salinity stress, wheat plants frequently devote more resources to root growth at the expense of shoot growth. To offset slower growth rates, some wheat cultivars postpone leaf senescence in order to sustain photosynthetic activity for longer [23]. The growth of thicker or more effective cuticles and surface structures to reduce water loss through evaporation may be what is meant by the references to “Slam term cuticles” and “Balanced cuticle vertical glades vessels” in Figure 1, whether by altering stomatal behavior or leaf architecture. In order to more effectively distribute nutrients to fewer, healthier branches, wheat plants may decrease the number of tillers. Shorter plants have shorter internodes, making them less vulnerable to mechanical stress and water loss. Certain types of wheat flower early in order to finish their life cycle before the effects of salinity stress become too great. To make sure that few nutrients are distributed among fewer, possibly more viable seeds, wheat may develop fewer spikelets. Phrases like “lower or smaller photosynthetic activity” and “minimizing water resources” suggest ways to save water, maybe by minimizing transpiration and increasing water usage efficiency (Figure 1). The presence of leaf hairs, or trichomes, is suggested by the phrase “Enhanced leaf hairs lower structure” as a way to prevent water loss and provide protection from environmental stress [21].

## 3. Mechanisms of Salinity Tolerance in Wheat

### 3.1. Physiological and Biochemical Responses to Salinity Tolerance in Wheat

**Figure 2:**
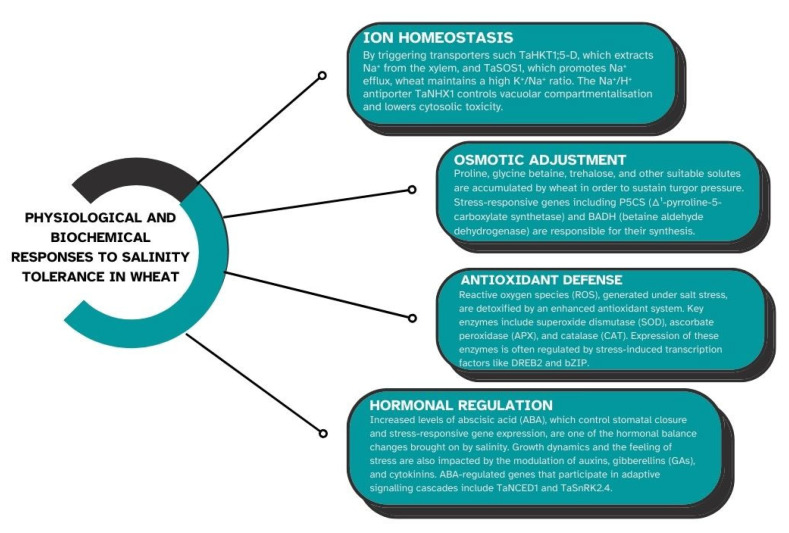
Physiological and biochemical responses to salinity tolerance in wheat.

A complicated web of physiological and metabolic processes controls wheat’s capacity to withstand salt stress (Figure 2). Wheat plants adjust in a variety of ways to preserve cellular homeostasis and lessen the negative impacts of salt [24]. Wheat plants have evolved a number of defenses against the damaging effects of salt stress. In order to maintain low sodium levels in the shoot, wheat plants can limit the net uptake of sodium at the soil–root interface and its xylem loading in the roots [25]. Wheat uses a number of physiological defense mechanisms against salt stress in order to withstand salinity. Ion homeostasis, using which wheat plants regulate the intake and distribution of potassium (K⁺) and sodium (Na⁺) ions to avoid toxicity and preserve cellular function, is one of the key mechanisms [26]. Another important mechanism is osmotic adjustment, in which wheat stores proline and glycine betaine, two compatible solutes, to hold onto water inside cells and stabilize proteins pursuant to stress. Furthermore, wheat reduces oxidative damage brought on by too much salt by activating antioxidant defense systems [27].

In order to create wheat varieties that are more resilient in salty soils, cultivation efforts must have an extensive awareness of these phenomena. By building up suitable solutes such proline, glycine betaine, and sugar alcohols, wheat plants can modify their cellular osmotic potential in addition to maintaining ion homeostasis. These osmolytes shield cellular structures from salt and aid them in preserving the water balance within cells [28]. Gaining insight into these processes lays the groundwork for creating tolerant cultivars. Furthermore, proline and glycine betaine, two compatible solutes that support cellular osmotic equilibrium and shield cellular structures in saline environments, can be accumulated by wheat plants [29].

#### 3.1.1. Ion Homeostasis

Salinity tolerance in wheat depends on ion homeostasis, which aids plants in controlling the ratio of toxic sodium (Na⁺) to helpful potassium (K⁺) ions, which is mentioned on Figure 2 [7]. Excess Na^+^ can build up in plant cells under salt stress, interfering with enzyme activity and causing toxicity. In order to counteract this, wheat employs certain transport proteins to restrict the uptake of Na^+^, separate it into vacuoles, or keep it out of cells. At the same time, it guarantees adequate K^+^ uptake to sustain cellular processes. Maintaining development and metabolic activity in saline environments requires an equilibrium between Na^+^ and K^+^. Maintaining ion homeostasis is a crucial factor for wheat’s ability to withstand salinity [28]. Overabundance of sodium (Na⁺) and chloride (Cl⁻) ions from saline soils can disrupt the intake of potassium (K⁺), which is necessary for stomatal regulation, osmotic balance, and enzymatic activities. Wheat plants use a variety of techniques to control ion toxicity:

- Selective Ion Uptake: To stop Na^+^ from building up in the cytoplasm, root cells preferentially absorb K^+^ over Na^+^.
- Exclusion of Na^+^: Salt-tolerant wheat cultivars either prevent Na^+^ from entering root cells or quickly export it via the plasma membrane’s Na^+^/H^+^ antiporters [30].
- Compartmentalization: By separating Na^+^ ions into vacuoles, wheat plants can prevent harmful cytoplasmic consequences.

#### 3.1.2. Osmotic Adjustment

In response to salinity stress, wheat plants can modify their cellular osmotic potential by building up suitable solutes such proline, glycine betaine, and sugar alcohols. These osmolytes shield cellular structures from salt and aid in preserving the water balance within cells [31]. The overexpression of genes involved in the manufacture of suitable solutes, such as betaine aldehyde dehydrogenase for glycine betaine and P5CS for proline, frequently mediates the accumulation of these solutes in wheat. One essential mechanism that enables wheat to tolerate salinity is osmotic adjustment, which maintains cell turgor and water balance under salt stress. Wheat cells acquire compatible solutes such as proline, glycine betaine, and sugars when exposed to high salinity; these solutes aid water retention without interfering with cellular processes [32]. In spite of the high concentration of salt outside, this mechanism lowers the osmotic potential within the cell, enabling it to absorb and retain water. Thus, osmotic adjustment allows wheat to continue growing and developing in saline soils by stabilizing proteins, preserving enzyme activity, and shielding cellular structures. Because salinity lowers the soil’s water potential, it makes it harder for roots to absorb water, which results in osmotic stress. Wheat plants store organic osmolytes such proline, glycine betaine, and carbohydrates to combat this. Under saline conditions, these osmolytes stabilize proteins and membranes, preserve cell turgor, and shield biological structures [33]. Proline and glycine betaine are examples of compatible solutes that wheat can accumulate to modify its osmotic potential. These solutes support the stabilization of proteins and cellular structures under osmotic stress, preserve cell turgor, and assist cells retain water. Osmotic stress brought on by salinity can result in a water shortage, which inhibits plant growth. By producing and storing suitable solutes including proline, glycine betaine, and carbohydrates, wheat plants use osmotic adjustment mechanisms to lessen this stress [34].

#### 3.1.3. Antioxidant Defense

Activation of signaling cascades that alter patterns of gene expression, the buildup of suitable solutes, and the enhancement of antioxidant defenses are some examples. By increasing the synthesis of plant growth regulators, which can initiate adaptive responses, wheat plants can also lessen the effects of salinity. Furthermore, research has demonstrated that wheat roots’ tissue-specific salt sequestration and accumulation are essential for their ability to withstand salinity [4]. Wheat’s antioxidant defense system is triggered in response to salinity stress in order to prevent oxidative damage from reactive oxygen species (ROS), which build up in environments with high salinity. In wheat, salinity stress can result in an excess of reactive oxygen species, which can harm cellular components through oxidative stress. To counteract the consequences of oxidative stress, wheat plants have developed a number of antioxidant defense mechanisms, including as the overexpression of genes encoding enzymes such ascorbate peroxidase, catalase (CAT), and superoxide dismutase (SOD) [35]. A key component of wheat’s ability to withstand salinity is the regulation of these antioxidant mechanisms. SOD, CAT, and peroxidase (POD) are important antioxidant enzymes that cooperate to neutralize ROS and stop damage to DNA, lipids, and proteins. Growth and survival in saline settings depend on wheat plants’ ability to tolerate and recover from salt-induced oxidative stress, which is made possible by this defense system, which also helps to preserve cellular health and function. The ROS, including hydrogen peroxide (H_2_O_2_) and superoxide radicals (O_2⁻_), are produced in response to salinity stress [36,37]. Cellular dysfunction can result from the harm that these ROS can do to proteins, lipids, and nucleic acids. Wheat cultivars that can withstand salt have stronger antioxidant defenses, which contain non-enzymatic antioxidants such ascorbate and glutathione as well as enzymes like glutathione peroxidase (GPX), CAT, and SOD [38]. Together, these compounds scavenge ROS and lessen oxidative damage. Wheat plants have developed a number of antioxidant defense mechanisms, such as the upregulation of genes encoding enzymes like superoxide dismutase, catalase, and ascorbate peroxidase, to counteract the effects of oxidative stress, which can result from the overproduction of reactive oxygen species caused by salinity stress [39].

#### 3.1.4. Hormonal Regulation

In order to balance development and stress responses, wheat’s reaction to salt stress is greatly influenced by hormonal control. To improve salt tolerance, important plant hormones such gibberellins, cytokinins (CK), auxins (IAA), and abscisic acid (ABA) modify a number of molecular processes. ABA levels rise in response to salt stress, which activates genes that respond to stress and cause stomatal closure to minimize water loss. Gibberellins control development, while other hormones, such as IAA and CK, modify growth patterns to preserve resources [40,41]. When combined, these hormonal changes allow wheat to modify its growth and survival tactics under salty conditions. Hormones from plants are essential for controlling wheat’s ability to endure salt. ABA plays a key role in inducing stress reactions such as osmolyte buildup, stomatal closure, and modifications in the expression of genes. Additionally, ethylene (ETH), jasmonic acid (JA), and salicylic acid (SA) mediate stress signaling pathways. Under stress, the hormonal crosstalk balances development and defiance processes to control plant growth [42]. Under stressful conditions, ABA decreases water loss, stimulates stress-responsive genes (DREB, LEA), causes stomatal closure, and improves osmotic adjustment. JA alters the antioxidant defense and root growth, increases the production of antioxidant enzymes (SOD, CAT), scavenges ROS, and lengthens roots. ETH encourages stress adaptation and root architecture, increases ROS elimination, boosts nutrient uptake, and controls root development. IAA controls cell division and signaling from root to shoot, stabilizes cell membranes, increases food and water intake, and improves root architecture. CK balances growth and stress responses, preserves cell division and postpones senescence in the face of adverse situations [40,41].

### 3.2. Gene Expression or Signal Transduction on Salinity Tolerance in Wheat

**Figure 3:**
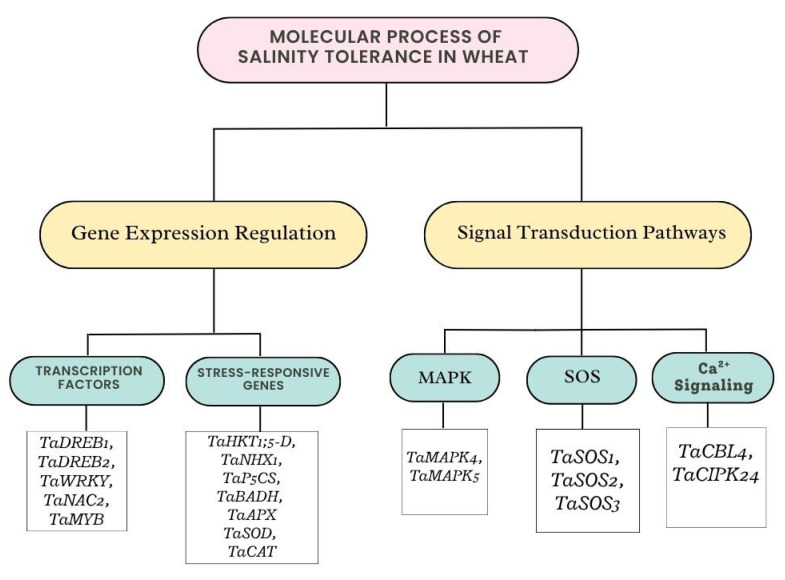
Molecular process of salinity tolerance in wheat resulting in gene expression or signal transduction.

Wheat is able to tolerate high salt levels attributable to a complex interaction between signal transduction pathways and gene expression, which allow for quick reactions to salt stress. The perception of salt stress and the transmission of the signal to start adaptive responses depend on signal transduction pathways [28]. Wheat’s salt tolerance mechanisms rely heavily on gene expression and signal transduction. Stress perception and signal amplification are significantly influenced by the salt overly sensitive (SOS) pathway, mitogen-activated protein kinase (MAPK) cascade, and calcium signaling. Stress-responsive gene expression is controlled by transcription factors such as DREB and NAC (Figure 3). The SOS pathway controls the exclusion of Na+ to maintain ion homeostasis. A Na^+^/H^+^ antiporter that exports Na+ from root cells is called SOS1. SOS1 is activated by a protein kinase, SOS2. A calcium sensor that communicates with SOS2 is called SOS3. Ion homeostasis is regulated by SOS pathways, of which SOS1 encodes a plasma membrane Na^+^/H^+^ exchanger, while SOS2 and SOS3 function as kinases that mediate ion transport in the presence of salt stress. Wheat perceives and reacts to salinity stress through intricate networks of transcriptional control and signaling channels [42]. The MAPK cascade controls gene expression and intensifies stress signals. It is triggered by salinity stress, which phosphorylates transcription factors that control genes that respond to stress [43]. A crucial signaling molecule in the plant’s reaction to salt stress is calcium signaling, which is serves as a backup messenger for stress reactions. The salt overly sensitive pathway is a well-established signaling cascade that controls ion homeostasis and improves wheat’s tolerance to salinity. It is triggered by calcium-binding proteins [44]. Certain signaling molecules, including calcium ions (Ca^2+^) and ROS, function as messengers when wheat plants sense salt stress, opening up pathways that result in the activation of genes that respond to stress [39]. The activation of calcium-binding proteins by the Ca^2+^ influx caused by salinity stress results in downstream signaling (Figure 3).

Certain genes that enable wheat to withstand high salt levels are expressed in response to salinity stress. Finding and using these genes for breeding is the goal of genomic research [45]. The associated proteins are encoded by genes. In order to preserve homeostasis and safeguard cellular structures in saline environments, transcription factors like DREB and NAC are essential for controlling these genes and coordinating the plant’s physiological reactions. Salinity tolerance is controlled by intricate gene networks that are involved in stress reactions and signal transduction [46]. Stress-responsive gene expression is modulated by transcription factors such the NAC (NAM, ATAF, and CUC) and DREB (Dehydration-Responsive Element Binding) families. It attaches to DRE/CRT elements found in stress-responsive gene promoters. Control genes related to development and stress tolerance and participate in stress reactions and ABA signaling [47]. It has been discovered that stress-responsive transcription factors, including DREB, MYB, and WRKY, are essential for triggering the expression of genes linked to salinity tolerance [44]. To create more resilient cultivars, it is essential to comprehend the regulatory processes that underlie wheat’s ability to endure salinity.

## 4. Genetic Management of Salt Tolerance in Wheat

### 4.1. Germplasm Evaluation and Screening for Salinity Tolerance in Wheat

To create salt-tolerant cultivars, wheat breeders have used a variety of techniques, such as germplasm screening, introducing genetic diversity, and using biotechnological methods. Significant genetic variation was found when wheat genotypes were screened for salinity tolerance; certain lines showed increased resistance to salt stress [4]. Numerous genetic resources have been identified as having enhanced tolerance to salt stress through extensive screening of wheat germplasm; these genetic resources can be used in breeding efforts to improve the salinity tolerance of commercial wheat cultivars. Because salinity tolerance in wheat is complex and polygenic, a multifaceted approach combining conventional breeding with these genetic resources is necessary [19]. Creating wheat cultivars that can flourish in highly salinized soils is known as salinity tolerance breeding. Current techniques like MAS allow breeders to target particular genes connected to salinity tolerance features, similarly to how traditional breeding selects inherently salt-tolerant lineages [48]. By adding genes that strengthen tolerance mechanisms, genetic engineering improves this even further. New methods, such as CRISPR gene editing, enable precise alterations to increase the resistance of wheat. In order to promote sustainable agriculture in regions affected by salt, these breeding techniques seek to create high-yield, salt-tolerant wheat types. Numerous conventional and contemporary methods have been used in breeding projects to increase salt tolerance [49,50]. The main strategy for creating wheat cultivars that salt has been to use selective breeding to take advantage of this natural variety. Furthermore, adding fungi that promote plant growth, like *Trichoderma longibrachiatum*, has the potential to increase wheat’s resistance to salinity. These advantageous microbes can alter gene expression, boost the antioxidant defense mechanisms, and enhance the general physiological function of wheat plants in salty environments [51,52]. To completely understand how these microbial interactions provide tolerance to salinity and to maximize their use in wheat breeding programs, more investigation is required. Developments in biotechnology, such as genome editing and genetic engineering, have also made it possible to create wheat cultivars that can endure salt. It has been shown that introducing genes related to oxidative stress management and salt exclusion can increase wheat’s resistance to salinity [53].

The main strategy for creating wheat cultivars that can endure salt is using selective breeding to take advantage of this natural variety [29,54]. In order to create offspring with increased resilience, conventional breeding for salinity tolerance in wheat entails choosing and crossing naturally salt-tolerant types. This approach depends on assessing characteristics such as ion balance, growth, and yield in saline environments. Breeders try to progressively increase salt tolerance in subsequent generations by crossing wheat lines with desired traits [55]. Even though it takes a lot of time, traditional breeding is still a key strategy for creating wheat that can endure salt, particularly when paired with field testing and selection in saline conditions. Because salinity tolerance features are typically polygenic, traditional breeding is difficult and time-consuming [56]. Nonetheless, it is frequently possible to introduce beneficial tolerance qualities into commercial cultivars of wheat from landraces and wild cultivars. The application of marker-assisted selection in wheat breeding programs has been made easier by the creation of molecular markers associated with salinity tolerance features. The creation of salt-tolerant wheat cultivars can be accelerated by pyramiding several tolerance-related features using these markers [29]. It improved resistance to salt stress by using genetic markers associated with salinity tolerance qualities. In comparison to traditional methods, MAS allows breeders to target and combine phenotypes quicker by locating and monitoring particular markers linked to salt tolerance in the genome [57]. Because it allows for an accurate selection and the integration of desired genes while preserving other crucial agronomic properties, MAS is very useful in the development of wheat varieties. It uses DNA markers associated with features related to salinity tolerance [58,59]. Numerous areas of the wheat genome have been linked to characteristic Na^+^ exclusion by quantitative trait loci (QTL) mapping. Using a variety of mapping populations and genetic platforms, a sizable number of quantitative trait loci linked to salinity tolerance in wheat have been found. In order to create salt-tolerant wheat cultivars, these QTLs can be used in genomic selective breeding programs and marker-assisted selection [60]. They also offer important insights into the genetic architecture of salinity tolerance. MAS makes it possible to choose tolerant lines quicker by integrating these markers into breeding strategies. One important characteristic for tolerance to salinity, Na^+^ exclusion, has been associated with QTLs on chromosome 2A. By focusing on genetic markers associated with salinity tolerance features, MAS speeds up the breeding process. Breeders can more effectively find and choose lines that can endure salt [61]. Wild wheat relatives and landraces are important genetic resources for improving salinity tolerance by introducing adaptive alleles. The effectiveness of germplasm screening for salinity tolerance is increased by advanced imaging techniques and physiological assessments. Wheat genotypes evaluated under controlled and field conditions aid in the identification of salt-tolerant lines based on traits such as biomass retention and yield stability [62].

### 4.2. Genetic Engineering for Salinity Tolerance in Wheat

The creation of salt-tolerant cultivars of wheat requires an understanding of the genetic underpinnings of salinity tolerance [63]. By preserving cell turgor and stabilizing proteins and cellular structures, these substances help plants hold onto water in salinized environments. Stress-responsive genes tightly control the manufacture of these osmolytes, underscoring the intricate relationship between genetics and environmental stress responses. To properly address this challenge, however, a multidimensional strategy combining traditional breeding, biotechnology, and integrated crop management techniques is required due to the polygenic nature of wheat’s salinity tolerance [64,65]. In order to develop more focused and effective breeding strategies, employing cutting-edge genomic and transcriptomic techniques can offer deeper insights into the intricate genetic engineering of wheat’s salinity tolerance [66,67]. When wheat genotypes were screened for salinity tolerance, a great deal of genetic variability was found, with some lines showing increased resistance to salt stress. Creating salt-tolerant wheat cultivars up until now mostly involved using selective breeding to take advantage of this inherent variance [52,68]. By directly introducing or altering genes involved in stress response, genetic engineering provides a potent method of increasing wheat’s resistance to salinity. In contrast to conventional breeding, genetic engineering enables the exact insertion of genes that are in charge of important tolerance mechanisms [69]. For instance, wheat can be made more tolerant to high-salt conditions by adding genes that improve sodium exclusion or encourage osmotic compensation. Therefore, genetic engineering offers a quicker and more focused method of creating wheat varieties, which is crucial for sustainable farming in saline soils. Novel approaches to introducing salinity tolerance features into wheat have been made possible by developments in genetic engineering [70]. Genetic engineering has been investigated in addition to traditional breeding. Wheat has benefited from the effective introduction of genes, which have increased growth and yield in saline environments. It is possible to overexpress or import genes from other species that are involved in ion transport [71,72]. For example, transgenic wheat that expresses the Arabidopsis AtNHX1 gene, which codes for a vacuolar Na⁺/H⁺ antiporter, has demonstrated enhanced resistance to salt stress. Furthermore, it is possible to precisely target and alter genes related to salt tolerance using CRISPR/Cas9 gene-editing technology. Newer tools like CRISPR/Cas9 gene editing and transgenic approaches allow for the modification of particular genes to increase salinity tolerance without compromising other agronomic properties [73].

One of the most important objectives for guaranteeing food security in the face of global salinization is the development of wheat cultivars that can endure salt. It may be possible to find the answers to this urgent problem by combining several genetic, genomic, and microbiological techniques [74]. Genomic selection (GS) is a sophisticated breeding technique that predicts an individual’s breeding value based on its genotype using genome-wide markers. By capturing the cumulative effect of small-effect loci, this method has proven effective in enhancing complex traits such as salinity tolerance [75]. Utilizing genome-wide markers, GS is a sophisticated breeding technique that predicts and chooses wheat seedlings with a high resistance to salinity. This approach determines the breeding value of complex traits, such as salt tolerance, controlled by several genes, by examining genes throughout the entire genome. Breeders can choose the best-performing plants early on without requiring a lot of field testing thanks to GS [76]. By concentrating on genetic potential rather than just observed features, this speeds up the breeding cycle and improves the effectiveness of creating salt-tolerant cultivars. It is an effective way to increase wheat’s resistance and productivity in salinized environments [77]. While genetic engineering directly introduces genes linked to salinity tolerance, GS selects tolerant types using genome-wide markers and predictive modeling. Additionally, CRISPR-based gene editing shows promise for specific enhancements in tolerance to salinity. Some wild cultivars of wheat, such species of *Aegilops*, have developed a remarkable resilience to salt stress and have adapted to flourish in extremely saline settings. Through backcrossing and interspecific hybridization, the genetic resources present in these halophytic wheat relatives can be introduced into farmed wheat to improve the cultivars [63,78]. There is growing evidence that the expression of wheat genes that are tolerant of salinity may also be regulated by epigenetic processes including DNA methylation and histone changes. There may be other ways to increase wheat’s resistance to saline conditions if the epigenetic foundation of salinity tolerance is understood [79], such as identifying genomic areas linked to salinity tolerance traits. By using high-density genotyping and association analysis, GWAS makes it possible to identify potential genes associated with tolerance to salinity. Understanding the gene expression, protein regulation, and metabolic pathways involved in salinity adaptation is possible through transcriptomics, proteomics, and metabolomics.

### 4.3. Traditional vs. Modern Breeding Techniques for Salinity Tolerance in Wheat

Advanced molecular and genomic techniques have gradually replaced conventional breeding methods in the search for wheat cultivars that can withstand salt. The development of salt-tolerant wheat varieties has benefited greatly from traditional breeding methods like phenotypic selection and hybridization; yet, these methods are sometimes constrained by the complex, polygenic nature of salinity tolerance and the significant environmental influences on trait expression [10]. Although these techniques work well for long-term selection, they are labor-intensive and imprecise. By focusing on certain genes or QTLs linked to salt tolerance, modern methods like CRISPR-Cas9 gene editing, GS, and MAS provide more accuracy and efficiency [7,48,80,81,82,83]. Some important studies related to salt tolerance improvement in wheat by using modern breeding techniques are shown in Table S1. However, the efficacy of MAS is restricted by inconsistent reported results and the scarcity of validated markers across a range of genetic origins [80]. Similar to this, although CRISPR-based strategies have demonstrated potential, their widespread use is constrained by regulatory issues, off-target effects, and a lack of field validations [81]. Furthermore, it is challenging to forecast performance in varied field situations because a large number of recent research are carried out in controlled settings [82]. These drawbacks show that in order to create resilient, salt-tolerant wheat cultivars appropriate for actual agricultural systems, integrative methods integrating genomic technologies with high-throughput phenotyping and multi-environment trials are required.

### 4.4. Case Studies of Salt-Tolerant Wheat Cultivars

Numerous case studies have shown how salt-tolerant wheat cultivars may be successfully developed and implemented, with both agronomic and financial benefits. For example, in India, cultivars with improved yield under salty conditions and enhanced sodium exclusion have been released as a result of the introduction of the Nax1 and Nax2 loci from *Triticum* monococcum into elite wheat backgrounds like KRL 213 and HD2009 via marker-assisted selection [7]. Another noteworthy instance is the transgenic wheat that overexpressed the *Arabidopsis thaliana* AtNHX1 gene, which improved vacuolar Na^+^ sequestration and permitted development in soils containing up to 250 mM NaCl [83]. More recently, the TaHKT1;5-D gene has been knocked out using CRISPR-Cas9 gene editing, which has improved root ion transport and increased tolerance to salinity in field tests [80]. Increased wheat output in marginally saline fields, less reliance on chemical inputs, and higher farmer incomes have all resulted from the adoption of such salt-tolerant varieties. Notwithstanding encouraging outcomes, regulatory restrictions on gene-edited and transgenic wheat, as well as limited field validation continue to be obstacles to wider adoption, highlighting the necessity of ongoing translational research and governmental support [83].

### 4.5. Role of High-Throughput Plant Phenotyping Technologies in Salt Tolerrance of Wheat

In recent years, high-throughput plant phenotyping (HTP) technologies, like hyperspectral imaging (HSI), remote sensing (RS), and machine learning (ML), have been widely used in plant morphological and physiological measurements in a non-destructive manner to enhance breeding processes [84,85]. The HSI system with satisfactory system setups can be adopted to estimate proximal sensing of nitrogen content in wheat with sufficient accuracy [86]. Camenzind and Yu [87] reported that the yield assessment across European wheat varieties can be conducted before flowering using multi temporal multispectral UAV remote sensing. Thus, changes in morphological and physiological parameters on wheat under salty conditions should be assessed with these HTP technologies. RGB imaging and spectral and thermal sensors are the most important sensing techniques for monitoring key morphological and physiological traits of plant salt tolerance [88]. There is a study highlighting the potential use of hyperspectral reflectance as a phenotyping tool for assessing salt tolerance in advanced spring wheat lines under field conditions, which would accelerate the development of salt-tolerant wheat genotypes in breeding programs [89]. Another research has regarded the application of ML in hyperspectral image analysis as a novel approach to estimate salt tolerance in wheat [90]. Additionally, thermal and RGB imaging combined with artificial neural networks are also recommended as a promising technique to assess the salt tolerance of wheat [91]. To sum up, HTP technologies will play crucial roles in the breeding process of salt-tolerant wheat genotypes.

## 5. Further Research

### 5.1. Identification of Novel Genes and Pathways

One of the most important abiotic factors affecting wheat development, productivity, and sustainability in dry and semi-arid regions of the world is soil salinity. Even though breeding methods and our understanding of salinity tolerance mechanisms have advanced significantly [92], the intricate relationships between physiological, molecular, and environmental factors remain unclear. Through interdisciplinary approaches, this proposal seeks to fill in the information gaps and offer a path for future wheat research on salinity tolerance. By examining genetic variants in various wheat populations, GWAS is used to find QTLs linked to significant salt tolerance features, such as antioxidant activity, osmotic adjustment, and Na^+^/K^+^ homeostasis. The gene expression profiles of wheat cultivars that are sensitive to and tolerant of salinity stress are compared using RNA sequencing, or RNA-Seq [93]. This aids in locating regulatory networks and differentially expressed genes. To ascertain their specific functions in salinity tolerance, uncharacterized genes discovered using GWAS or transcriptomics are further investigated utilizing gene knockout/knockdown techniques (such as CRISPR-Cas9 or RNA interference). The function of miRNAs, which post-transcriptionally control gene expression, in focusing on stress-related pathways is examined [63]. These methods are employed to identify new stress-related proteins and metabolites involved in ROS detoxification, osmolyte production, or ion homeostasis. In light of worldwide salinization, creating wheat cultivars that can withstand salt is an essential objective for maintaining food security. The key to solving this urgent problem may lie in combining several genetic, genomic, and microbiological techniques with a deeper comprehension of the underlying physiological and molecular pathways [53].

### 5.2. Hormonal Cross-Talk Studies

In wheat, resistance to salinity is frequently linked to resistance to other abiotic stimuli including heat and drought. Developing wheat cultivars that are robust and resilient requires taking into account the possible trade-offs and interactions with these additional stressors when breeding for increased salinity tolerance [24]. The goal of hormonal cross-talk research is to comprehend how plant hormones interact and synchronize wheat’s reactions to salinity stress by investigating histone alterations in stress-responsive genes using chromatin immunoprecipitation sequencing. The DNA methylation patterns in wheat lines that are sensitive to and tolerant to salinity are mapped using whole-genome bisulfite sequencing. An analysis of how stress memory, or epigenetic priming, can help wheat become more tolerant of salinity is conducted [94,95]. To determine their functions in stress signaling and adaptation, researchers measure changes in the levels of hormones such as IAA, CK, SA, ETH, JA, and ABA during salinity stress. examining the ways in which pathways combine to modify physiological responses such as ion homeostasis, osmotic adjustment, and antioxidant defense (e.g., ABA-ethylene signaling in stomatal regulation or JA-SA interactions in ROS detoxification). The role of certain hormones and their interactions under salt stress by using wheat lines with impaired hormonal signaling (such as ABA-deficient or ethylene-insensitive mutants) is identified and signaling networks that trace the regulatory relationships of stress-responsive genes and hormones are built in order to find master regulators [96,97].

### 5.3. Genome Editing for Salinity Tolerance

The wheat plant’s adaptive responses to salinity stress also heavily depend on the control of plant growth regulators such abscisic acid. The aim of genome editing for salinity tolerance is to accurately alter genes important to wheat responses to salinity stress by employing cutting-edge technologies such as CRISPR/Cas9. Using phenotyping tools (such as drones and hyperspectral imaging), biochemical and physiological characteristics of wheat exposed to salinity stress are tracked [53,98]. The genotype–environment interactions are assessed by evaluating performance in multi-environment trials. Salinity tolerance can be predicted using machine learning models based on DNA and phenotypic data. To improve salt tolerance characteristics, including ion homeostasis and osmotic adjustment, genes like SOS_1_ (sodium efflux), HKT1;5 (sodium transport), and transcription factors like DREB and WRKY can be edited [99]. To maximize the production of stress-responsive genes under salt stress, base editing or prime editing can be used to change regulatory areas or introduce point mutations in promoter regions. Mutants with better Na^+^/K^+^ equilibrium or increased antioxidant enzyme activity are created to lessen oxidative and ionic damage brought on by salt. To make sure the modifications increase tolerance without compromising yield or other agronomic characteristics, edited wheat lines are examined in both lab and field settings. New candidate genes, QTLs, and pathways involved in tolerance to salinity are discovered [100]. Thorough comprehension of the interplay between hormones and epigenetic control in wheat during salt stress is needed. Wheat cultivars with better yield stability in salty soils are created and research results are applied to improve the resistance of other crops and cereals to salinity stress. The wheat cultivars resistant to salt are created by using cutting-edge genome-editing technology. Field-level salinity tolerance screening procedures are improved with high-throughput instruments. The breeding initiatives can be accelerated by combining phenotypic, physiological, and molecular data [101,102].

## 6. Conclusions

Physiological, biochemical, and molecular processes all play a part in wheat’s complex and multidimensional tolerance to salinity (Table S2). Gaining knowledge of these processes—which include ion homeostasis, osmotic adjustment, antioxidant defense, hormonal control, and stress-responsive gene expression—is essential to understanding how wheat plants react to salinity. Using cutting-edge breeding techniques like CRISPR-Cas9 gene editing, genomic tools, and marker-assisted selection has greatly improved the capacity to create wheat types that can withstand salinity. Finding and using genetic resources to increase salinity tolerance is further accelerated by high-throughput phenotyping and multi-omics technology. Despite these developments, there are still issues, like the polygenic nature of salt tolerance, the need for field confirmation of lab results, and the trade-off between yield and stress tolerance. Investigating the genetic variety of wild wheat relatives, understanding epigenetic regulation, and combining multi-omics data to find new targets for genetic improvement should be the main goals of future study. Researchers, breeders, and legislators must work together to convert these scientific discoveries into workable solutions for farmers. In order to ensure sustainable wheat production in the face of rising soil salinization and climate change, the development of salinity-tolerant wheat varieties can be hastened by fusing conventional breeding techniques with state-of-the-art biotechnological tools. This review emphasizes the need of multidisciplinary strategies in tackling the worldwide problem of salt stress and the ways in which contemporary science may support agricultural resilience and food security.

## Data Availability

Data are contained within the article and Supplementary Materials.

## Supplementary Materials

The following supporting information can be downloaded at: https://www.mdpi.com/article/10.3390/plants14111641/s1, Table S1: Modern breeding techniques for salt tolerance improvement in wheat; Table S2: Salt tolerance mechanisms in physiological, biochemical and molecular responses in wheat.
