# Supplementary material for: Salinity Tolerance in Wheat: Mechanisms and Breeding Approaches
Source: Plants (Basel). 2025 May 27;14(11):1641. doi: 10.3390/plants14111641 (PMC12158024; doi:10.3390/plants14111641)
Supplement: Supplementary file 1 [file plants-14-01641-s001.zip › plants-3610722-supplementary.pdf]

Supplementary Tables

Table S1. Modern breeding techniques for salt tolerance improvement in wheat.

Modern breeding technique	Wheat line/genotype	Salinity tolerance traits	Associated elements (Genes/hormones/transporters)	References
Genomic tools (QTL mapping)	Seri M82 × Babax RIL population	Improved seedling vigor and biomass under salt stress	QTLs on chromosomes 2A, 3B, 4D; associated with ion balance and biomass	[80]
CRISPR-Cas9 gene editing	Modified wheat (TaHKT1;5-D KO)	Increased Na^+ exclusion; improved ion homeostasis	TaHKT1;5-D (Na^+ transporter); SOS1; lower Na^+ accumulation in shoots	[81]
Genomic selection (GS)	Elite breeding lines (unnamed)	Higher grain yield and survival under salt conditions	Genomic-estimated breeding values (GEBVs) for salinity-related traits	[82]
Marker-assisted selection (MAS)	Kharchia 65 × HD2009 (derived line)	Enhanced K^+/Na^+ ratio; better grain yield under salinity	Nax1 and Nax2 QTLs linked to Na^+ exclusion	[7]
Transgenic (Gene overexpression)	*AtNHX1*-expressing wheat	Higher salt tolerance; increased vacuolar Na^+ sequestration	AtNHX1 (Arabidopsis Na^+/H^+ antiporter gene)	[83]

Table S2. Salt tolerance mechanisms in physiological, biochemical and molecular responses in wheat.

Tolerance mechanisms	Basic descriptions	Key components	Functions in salt tolerance	References
Ion homeostasis	Controls the absorption, movement, and segregation of harmful ions (such as Na^+)	*HKT1;5*, *SOS1*, *NHX1*, *Nax1*, *Nax2*, plasma membrane ATPases	Maintains low cytosolic Na^+, high K^+/Na^+ ratio	[42,80,83]
Osmotic adjustment	Solute accumulation to preserve cell turgor and hold onto water	Proline, glycine betaine, sugars, aquaporins	Protects cells from dehydration due to osmotic stress	[9,35]
Antioxidant defense	Reactive oxygen species (ROS) generated under stress are scavenged	SOD, CAT, POD, ascorbate, glutathione	Prevents oxidative damage to cellular structures	[35–39]
Hormonal regulation	Signaling molecules are used to modify the stress response	ABA, ethylene, jasmonic acid, auxins	Regulates stomatal closure, gene expression, and growth arrest	[40–42]
Calcium signaling & SOS pathway	Early detection and communication of salt stress via the SOS cascade and Ca^{2+} influx	*SOS1*, *SOS2*, *SOS3*, CBLs, CDPKs	Triggers downstream tolerance pathways like ion transport	[81]
Gene expression regulation	Transcriptional networks that regulate the stress response being activated	*DREB*, *NAC*, *bZIP*, miRNAs	Induces stress-responsive genes, enhances adaptive traits	[32]
